# Association between dietary inflammation index and cataract: a population-based study from NHANES 2005–2008

**DOI:** 10.3389/fnut.2024.1379317

**Published:** 2024-04-04

**Authors:** Yi Zhang, Jingxin Zhou, Wenyue Shen, Andrzej Grzybowski, Kai Jin, Juan Ye

**Affiliations:** ^1^Eye Center, The Second Affiliated Hospital, School of Medicine, Zhejiang University, Hangzhou, China; ^2^Department of Ophthalmology, The First Affiliated Hospital of Zhejiang Chinese Medicine University, Hangzhou, China; ^3^Institute for Research in Ophthalmology, Foundation for Ophthalmology Development, Poznan, Poland

**Keywords:** cataract, dietary inflammation index, nutrition, epidemiology, dietary recall

## Abstract

**Importance:**

Various studies have widely explored the association between index of dietary inflammation (DII) and occurrence of diseases. Accumulating evidence have revealed that a lower DII seems to be protective against a variety of diseases. Nevertheless, the association between DII and age-related cataract remains unclear.

**Objective:**

To investigate the correlation between DII and age-related cataract in a representative sample of the American population.

**Design, setting, and participants:**

This cross-sectional population-based study comprised 6,395 participants from the National Health and Nutrition Examination Survey (NHANES) conducted in cycles from 2005 to 2008. DII was calculated using dietary recall information, with higher scores indicating greater inflammatory potential of the diet. Age-related cataract was evaluated using cataract surgery as a surrogate measure. Covariates included sociodemographic factors, lifestyle factors, physical measures, and comorbidities. Logistic regression models were employed to assess the association between DII and cataract. The presence of a non-linear relationship was examined using restricted cubic spline analysis. Subgroup analysis was conducted to explore potential interaction effects. Data analysis was performed from September 1 to December 30, 2022.

**Main outcomes and measures:**

Age-related cataract assessed through cataract surgery information obtained from a self-reported questionnaire.

**Results:**

A total of 6,395 participants were included, with a mean (standard deviation, SD) age of 48.7 (15.3) years. Of these, 3,115 (48.7%) were male, 3,333 (52.1%) were non-Hispanic white, and 683 (10.7%) had cataract. The mean (SD) DII was −4.78 (1.74). After adjusting for all included covariates, DII showed a positive association with cataract, both as a continuous variable (odds ratio (OR): 1.054, 95% confidence interval (CI): 1.007–1.103, *p* = 0.023) and in quartiles, with the highest quartile compared to the lowest (OR: 1.555, 95% CI: 1.233–1.967, *p* < 0.001). Restricted cubic spline analysis revealed no evidence of a non-linear relationship (*p* for non-linearity 0.085). Subgroup analysis indicated no interaction effects among the studied covariates.

**Conclusions and relevance:**

These findings suggest that a pro-inflammatory diet serves as a risk factor for the occurrence of cataracts.

## 1 Introduction

Cataract remains a globally prevalent cause of vision impairment and blindness among the elderly population, presenting a substantial public health concern around the world (1). In the United States, cataract prevalence rises from approximately 24.4% in those aged 40 and older to over 50% in individuals aged 75 and above, highlighting a significant increase with advancing age. The clouding of lenses, often attributed to aging, is a primary characteristic of age-related cataract, influenced by multifactorial determinants including lifestyle and environmental factors such as smoking, alcohol consumption, and prolonged exposure to sunlight (2–5). Despite the efficacy of cataract surgery in restoring vision, its widespread accessibility is hindered by economic constraints and inadequate surgical resources, particularly in developing nations (6). Addressing modifiable risk factors assumes paramount importance in mitigating the health and socioeconomic burden associated with cataract (7).

Inflammation plays a pivotal role in the development of several chronic diseases, including cardiovascular conditions (8) and certain cancers (9), and has been posited as a potential mechanism in age-related cataract. Concerning dietary patterns and overall diet quality have been associated with health outcomes, recent years have witnessed significant attention toward the Dietary Inflammation Index (DII) (10). The DII is computed based on the potential impact of foods and nutrients on the body’s inflammatory levels (11). While chronic inflammation might play a role in the pathogenesis of cataracts, research exploring the association between the DII and cataract incidence remains limited. The DII is derived from hundreds of foods collected globally and their impact on inflammatory levels (12). Higher DII scores indicative of elevated levels of chronic inflammation, suggesting a less healthy diet (13), a state that has been associated with an increased risk of age-related cataracts.

In this study, we aim to utilize data from the National Health and Nutrition Examination Survey (NHANES) conducted between 2005 and 2008 to investigate the relationship between DII and cataract development. We hypothesize that elevated DII scores are associated with a heightened risk of developing cataracts. By integrating both measures of healthy dietary patterns and inflammatory indices, we aim to comprehensively assess the impact of dietary patterns on cataract risk, providing potential new nutritional intervention strategies for cataract prevention and management.

## 2 Methods

### 2.1 Study population

NHANES, a substantial and nationally inclusive survey, are crafted to evaluate the health and nutritional condition of the American population. This survey is conducted by the National Center for Health Statistics at the U.S. Centers for Disease Control and Prevention (14). NHANES data is systematically structured in a biannual format, which is a publicly accessible resource in the United States.

We analyzed data from two consecutive National Health and Nutrition Examination Survey (NHANES) cycles (2005–2006 and 2007–2008). This analysis builds upon findings from our most recent study, which demonstrated that adherence to the Healthy Eating Index-2015 is associated with a reduced risk of age-related cataracts (15). By extending our investigation to include inflammatory dietary patterns through the DII, we aim to further elucidate the relationship between diet and cataract risk, enhancing our understanding of potential preventive nutritional strategies. Of all 20,497 participants in NHANES 2005–2008, we excluded those without complete information on cataract (*N* = 9,592) and diet (*N* = 973). Further, we excluded participants under 30 years old (*N* = 1,446) and without complete information on other covariates (*N* = 2091). Finally, 6,395 subjects were included in the analytic population.

### 2.2 Cataract definition

Cataract operation was used as a surrogate for cataract. Cataract operation was determined by asking participants the question “Have you ever had a cataract operation?” (VIQ071), with answers “yes” or “no.” If the answer is “yes,” the participant was diagnosed with cataract.

### 2.3 Dietary inflammation index calculation

Determination of the DII involved the acquisition of dietary intake details within the NHANES framework. These particulars were gathered through 24 h dietary recall sessions administered at the Mobile Examination Center. Oversight and implementation of the dietary data collection methodology, database maintenance, and data review were managed by the Food Surveys Research Group within the US Department of Agriculture. The DII’s development and validation, as documented by Shivappa et al. (16, 17), were instrumental.

The derivation of DII scores hinged upon the initial 24 h dietary data. Our investigation drew from 27 food parameters accessible in the NHANES database, encompassing various nutrients and components such as proteins, fats, carbohydrates, alcohol, fiber, cholesterol, omega-3 and omega-6 polyunsaturated fatty acids, among others. Each parameter corresponded to an assigned inflammatory effect score, elucidated meticulously in the Supplementary material. Methodologies for computing the DII were explicitly detailed.

Calculation of the DII involved scaling each food component per 1,000 calories consumed, referred to as the energy-adjusted dietary inflammatory index (E-DII) (18). This composite score encapsulated the anti-inflammatory (reflected in low DII scores) to pro-inflammatory (evidenced by high DII scores) properties manifest in the participant’s dietary habits.

### 2.4 Covariates

Sociodemographic factors were drawn from self-reported questionnaires, including gender, age, race, education, marital status, economic situation (family income poverty ratio <1.00, or ≥1.00). BMI was calculated as weight (kg) divided by height squares (m^2^) using information from body measurement examinations and further categorized into 3 classes (<18.5, 18.5–25, >25 kg/m^2^).

Lifestyle factors were obtained from self-reported questionnaires. Alcohol usage was calculated and categorized as lifetime abstainer, former drinker, current drinker ≤3 drinks/week and current drinker >3 drinks/week. Smoking was divided into 3 categories: never smoker, former smoker and current smoker.

Comorbidities studied in this study included hypertension, hyperlipidemia and diabetes mellitus. Participants were considered to have hypertension if they had been told by their doctors that they had hypertension, or they were taking anti-hypertension drugs, or systolic blood pressure was 140 mmHg or greater, or diastolic blood pressure was 90 mmHg or greater. Diagnosis of hyperlipidemia was made if participants were told they had hyperlipidemia, or taking cholesterol-lowering drugs, or total cholesterol was no less than 240 mg/dL during NHANES blood test. Presence of diabetes mellitus was determined if participants were told they had diabetes mellitus, or taking glucose-lowering drugs, or using insulin injection, or glycosylated hemoglobin (%) was 6.5% or greater during the NHANES test.

### 2.5 Statistical methods

Continuous variables were described using mean standard deviation (SD), and categorical variables were presented as numbers and percentages. DII was analyzed both as continuous and categorical variables based on quartiles. Variables were compared using Student’s *t*-test, or Rao-Scott Pearson *χ*^2^ test. To investigate the association between DII and cataract, three logistic regression models were established. Restricted cubic spline model with 4 knots was utilized to explore potential non-linear associations. Subgroup analyses based on covariates were conducted to investigate differences between subgroups and explore latent interaction effects.

The statistical analysis and visualization were conducted using R (version 4.1.1, R Foundation for Statistical Computing, Vienna, Austria). All statistical tests were two-tailed with a *p*-value of 0.05 or smaller as significant.

## 3 Results

### 3.1 Study population characteristics

A total of 6,395 individuals were enrolled in the study cohort, with an average age of 48.7 years. Among them, 3,115 (48.7%) were identified as male, and 3,280 (51.3%) as female. Table 1 provides a summary of their characteristics. Individuals who underwent cataract surgery exhibited a higher likelihood of being female, older, less educated, unmarried, and enjoying a relatively more favorable family economic status. The presence of a history of smoking or alcohol use was associated with an increased probability of cataract development. Patients diagnosed with hypertension, hyperlipidemia, and diabetes mellitus also faced an elevated risk of cataract formation. Table 1 reveals that participants with cataract tended to possess higher DII scores, agreeing with our initial hypothesis. Further investigation through multivariate analysis is warranted.

**Table 1:** Characteristics of participants stratified by cataract from NHANES 2005–2008.

	All	Non-cataract	Cataract	*p*-value
Number	6,395	5,712 (89.3)	683 (10.7)	
Gender (*N*, %)				0.034
Male	3,115 (48.7)	2,809 (49.2)	306 (44.8)	
Female	3,280 (51.3)	2,903 (50.8)	377 (55.2)	
Age [years, mean (SD)]	48.7 (15.3)	51.7 (14.1)	74.4 (9.0)	<0.001
Race (*N*, %)				<0.001
Non-Hispanic White	3,333 (52.1)	2,840 (49.7)	493 (72.2)	
Non-Hispanic Black	1,345 (21.0)	1,258 (22.0)	87 (12.7)	
Mexican American	1,061 (16.6)	1,009 (17.7)	52 (7.6)	
Other	656 (10.3)	605 (10.6)	51 (7.5)	
Education (*N*, %)				<0.001
Less than high school	1712 (26.6)	1,476 (25.8)	236 (34.6)	
High school or above	4,683 (73.4)	4,236 (74.2)	447 (65.4)	
Marital status (*N*, %)				<0.001
Unmarried or other	2,236 (35.0)	1911 (33.5)	325 (47.6)	
Married or living with partner	4,159 (65.3)	3,801 (66.5)	358 (52.4)	
Poverty (*N*, %)				<0.001
Below poverty	1,018 (15.9)	933 (16.3)	85 (12.4)	
Poverty or above	5,377 (84.1)	4,779 (83.7)	598 (87.6)	
BMI (*N*, %)				0.018
<18.5	83 (1.3)	75 (1.3)	8 (1.2)	
18.5 ~ 25	1,617 (25.3)	1,414 (24.8)	203 (29.7)	
≥25	4,695 (73.4)	4,223 (73.9)	472 (69.1)	
Alcohol usage (*N*, %)				<0.001
Lifetime abstainer	970 (15.2)	805 (14.1)	165 (24.2)	
Former drinker	1,079 (16.9)	905 (15.8)	174 (25.5)	
Current drinker ≤3 drinks/week	2,686 (42.0)	2,473 (43.3)	213 (31.2)	
Current drinker >3 drinks/week	1,660 (26.0)	1,529 (26.8)	131 (19.2)	
Smoking (*N*, %)				<0.001
Never smoke	3,305 (51.7)	2,973 (52.0)	332 (48.6)	
Former smoker	1842 (28.8)	1,550 (27.1)	292 (42.8)	
Current smoker	1,248 (19.5)	1,189 (20.8)	59 (8.6)	
Hypertension (*N*, %)				<0.001
No	3,122 (48.8)	2,958 (51.8)	164 (24.0)	
Yes	3,273 (51.2)	2,754 (48.2)	519 (76.0)	
Hyperlipidemia (*N*, %)				<0.001
No	3,566 (55.8)	3,262 (57.1)	304 (44.5)	
Yes	2,829 (44.2)	2,450 (42.9)	379 (55.5)	
Diabetes mellitus (*N*, %)				<0.001
No	5,370 (84.0)	4,880 (85.4)	490 (71.7)	
Yes	1,025 (16.0)	832 (14.6)	193 (28.3)	
DII [mean (SD)]	−4.78 (1.74)	−4.80 (1.75)	−4.64 (1.67)	0.023
DII quartile (*N*, %)				
Q1	1,599 (25.0)	1,468 (25.7)	131 (19.2)	<0.001
Q2	1,599 (25.0)	1,410 (24.7)	189 (27.7)	
Q3	1,597 (25.0)	1,429 (25.0)	168 (24.6)	
Q4	1,600 (25.0)	1,405 (24.6)	195 (28.6)	

*SD, standard deviation; BMI, body mass index; DII, dietary inflammation index; Q, quartile.

### 3.2 Association of DII and cataract using logistic regression

Results of logistic regression models for examining the relationship between DII and cataract are presented in Table 2. In the non-adjusted model (Model 1), DII showed a positive association with cataract, both as a continuous variable (OR: 1.054, 95% CI: 1.007–1.103, *p* = 0.023) and as a categorical variable in the highest quartile compared to the lowest (OR: 1.555, 95% CI: 1.233–1.967, *p* < 0.001). In the meantime, results in the minimally adjusted model (Model 2) and fully adjusted model (Model 3) displayed a similar trend. DII exhibited significant promotive effects against cataract in Model 2 (continuous variable: OR 1.062, 95% CI 1.004–1.124, *p* = 0.035; quartile: OR 1.338, 95% CI 1.012–1.774, *p* = 0.042) and Model 3 (continuous variable: OR 1.060, 95% CI 1.002–1.122, *p* = 0.043; quartile: OR 1.324, 95% CI 1.010–1.746, *p* = 0.045). Consistent with univariate regression, multivariate regression models provide more reliable insights. Namely, DII is positively associated with cataract in this study. Nonetheless, further analysis is necessary to resolve the apparent contradiction.

**Table 2:** Association of DII with cataract.

	Model 1^a^	Model 2^b^	Model 3^c^
DII	1.054 (1.007–1.103), 0.023	1.062 (1.004–1.124), 0.035	1.060 (1.002–1.122), 0.043
DII quartile			
Q1	reference	reference	reference
Q2	1.502 (1.190–1.189), 0.001	1.242 (0.940–1.645), 0.128	1.252 (0.952–1.651), 0.109
Q3	1.317 (1.037–1.676), 0.024	1.322 (0.993–1.762), 0.057	1.231 (0.932–1.628), 0.143
Q4	1.555 (1.233–1.967), <0.001	1.338 (1.012–1.774), 0.042	1.324 (1.010–1.746), 0.045

*DII, dietary inflammation index; Q, quartile.

a Non-adjusted model adjusted for: none.

b Minimally-adjusted model adjusted for: gender, age, race.

c Fully-adjusted model adjusted for all covariates.

### 3.3 Investigation of non-linear association using restricted cubic spline

**Figure 1:**
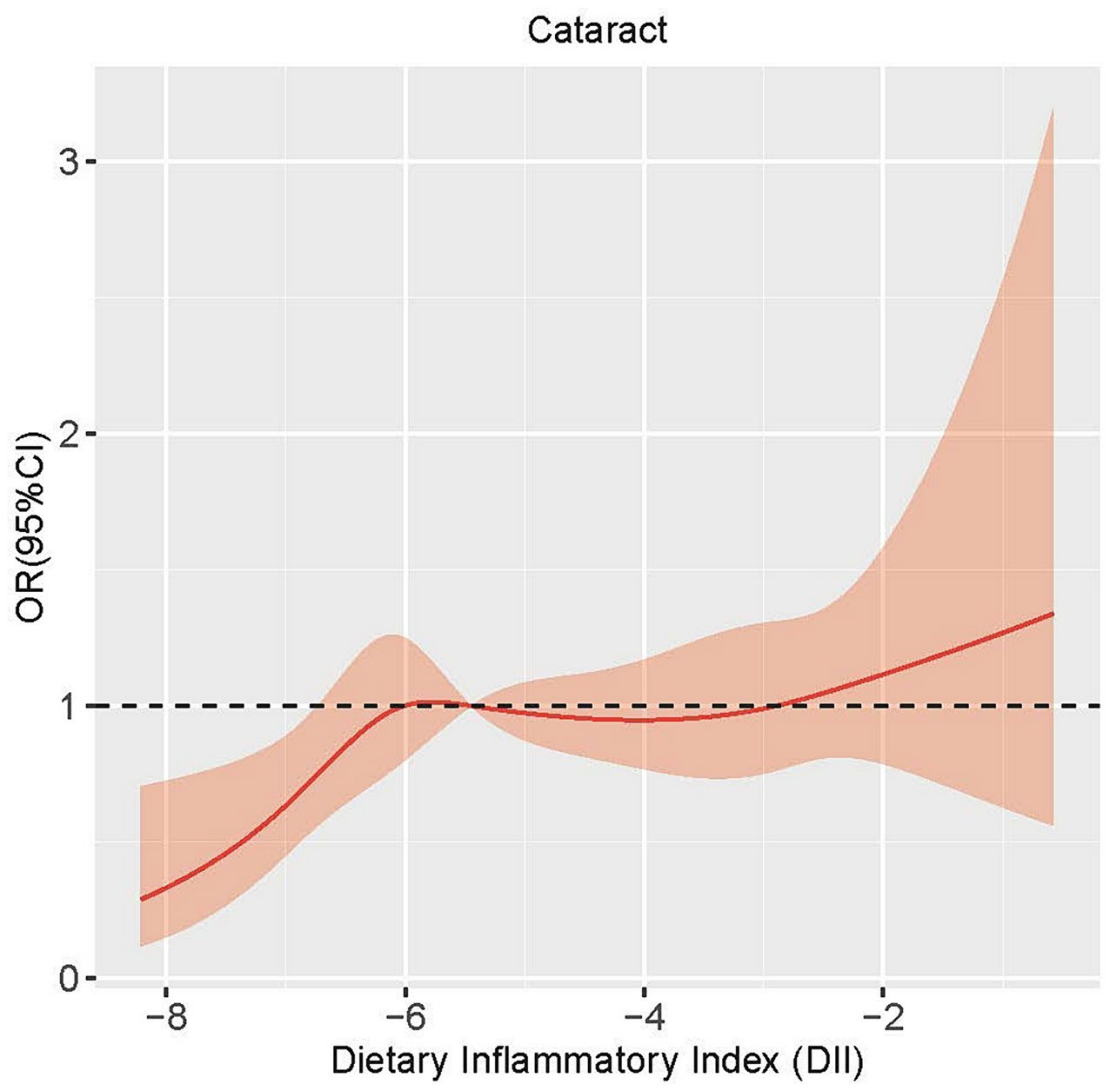
Restricted cubic spline analysis of the association between DII and cataract.

In order to assess the potential existence of a nonlinear relationship between DII and cataract, we employed a 4-knot restricted cubic spline. The *p*-value for the non-linearity test was 0.085, signifying the absence of a statistically significant nonlinear correlation between DII and cataract. As shown in Figure 1, the curve illustrates a general increasing trend, suggesting a positive correlation between DII and the development of cataract.

### 3.4 Subgroup analysis

Subgroup analyses were conducted on all covariates using the fully adjusted logistic regression model, and the results are summarized in Table 3. For most participant groups, DII remained a risk factor against cataract. Even among participants with hypertension, hyperlipidemia, and diabetes mellitus, DII retained its promotive association.

**Table 3:** Subgroup analysis of the association of DII with cataract.

		DII as continuous variable	DII as quartiles (Q1 as reference)		
			Q2	Q3	Q4
Age (years)	30–50	1.277 (0.953–1.750), 0.109	0.803 (0.104–4.941), 0.812	1.658 (0.399–8.200), 0.494	2.249 (0.530–1.133), 0.281
	50–70	1.019 (0.926–1.121), 0.696	1.207 (0.735–2.000), 0.460	1.389 (0.855–2.285), 0.188	1.186 (0.720–1.746), 0.504
	>=70	1.065 (0.994–1.141), 0.073	1.338 (1.012–1.774), 0.376	1.324 (1.010–1.746), 0.246	1.324 (1.010–1.746), 0.115
Gender	Male	1.083 (0.999–1.174), 0.052	1.214 (0.821–1.799), 0.330	1.249 (0.841–1.860), 0.271	1.452 (0.977–2.162), 0.065
	Female	1.032 (0.951–1.119), 0.455	1.143 (0.754–1.744), 0.530	1.286 (0.838–1.985), 0.253	1.177 (0.781–1.786), 0.439
Ethnicity	Non-Hispanic White	1.054 (0.986–1.128), 0.124	1.178 (0.845–1.646), 0.333	1.277 (0.912–1.792), 0.154	1.324 (0.946–1.856), 0.102
	Non-Hispanic Black	1.011 (0.739–2.330), 0.879	1.431 (0.678–3.140), 0.356	1.074 (0.487–2.424), 0.860	1.207 (0.565–2.669), 0.632
	Mexican	0.967 (0.809–1.151), 0.707	0.654 (0.252–1.612), 0.364	0.595 (0.246–1.404), 0.239	0.698 (0.305–1.591), 0.390
	Others	1.169 (0.960–1.430), 0.123	4.757 (1.423–20.217), 0.019	3.832 (1.150–16.094), 0.042	5.407 (1.626–22.876), 0.011
BMI (kg/m^2^)	<25	1.100 (0.988–1.226), 0.083	1.714 (1.002–2.959), 0.050	1.190 (0.670–2.118), 0.553	1.961 (1.157–3.366), 0.013
	>=25	1.033 (0.969–1.102), 0.322	1.114 (0.804–1.546), 0.517	1.227 (0.888–1.699), 0.216	1.154 (0.831–1.604), 0.392
Hypertension	Non- Hypertension	1.122 (1.008–1.251), 0.035	1.065 (0.609–1.860), 0.128	1.378 (0.803–2.370), 0.109	1.745 (1.039–2.954), 0.109
	Hypertension	1.031 (0.967–1.099), 0.352	1.303 (0.945–1.802), 0.107	1.197 (0.864–1.664), 0.280	1.226 (0.886–1.702), 0.220
Hyperlipidemia	Non-Hyperlipidemia	1.069 (0.985–1.161), 0.109	1.149 (0.759–1.744), 0.512	1.221 (0.803–1.860), 0.352	1.447 (0.964–2.179), 0.075
	Hyperlipidemia	1.030 (0.957–1.108), 0.436	1.294 (0.895–1.881), 0.173	1.240 (0.852–1.810), 0.263	1.230 (0.847–1.796), 0.278
Diabetes mellitus (DM)	Non-DM	1.051 (0.986–1.121), 0.130	1.301 (0.942–1.780), 0.111	1.254 (0.906–1.739), 0.173	1.332 (0.966–1.843), 0.082
	DM	1.037 (0.933–1.152), 0.498	1.052 (0.614–1.816), 0.854	1.159 (0.667–2.028), 0.601	1.222 (0.714–2.111), 0.467

## 4 Discussion

In this cross-sectional study, we investigated the link between DII and cataract prevalence, using data from 6,395 participants in a national survey. Findings showed a notable risk relationship between DII and cataract surgery, suggesting that DII promotes against cataract development. Additional analyses, such as propensity score weighted regression, non-linear tests, and subgroup analysis, supported this conclusion.

The DII is a comprehensive tool designed to quantify the inflammatory potential of an individual’s diet (19). It takes various dietary components into account and has emerged as a valuable instrument for assessing the inflammatory aspects of dietary patterns. Understanding the relationship between DII and chronic inflammatory diseases is crucial for advancing our knowledge of preventive measures and therapeutic interventions (20). A number of studies have indicated that a pro-inflammatory diet, as reflected by a higher DII, is associated with an increased risk of developing cardiovascular conditions (21, 22), developing insulin resistance and impaired glucose metabolism (23), type 2 diabetes (24), various cancers (25–28).

While current research provides valuable insights into the association between DII and chronic inflammatory diseases, certain challenges exist. Variability in study designs, dietary assessment methods, and outcome measures create significant hurdles in comparing results across studies (29). Additionally, the complex interplay of genetic, environmental, and lifestyle factors requires further investigation to delineate the precise mechanisms linking DII to chronic inflammatory diseases. Future research endeavors should focus on prospective cohort studies, employing standardized methodologies to enhance comparability. Mechanistic studies exploring the immunomodulatory effects of dietary patterns will deepen our understanding of the biological underpinnings of DII-related health outcomes. Ultimately, a comprehensive understanding of the relationship between DII and chronic inflammatory diseases holds promise for developing targeted dietary interventions to mitigate the burden of these conditions.

Cataract, a leading cause of visual impairment globally, is characterized by the clouding of the eye’s natural lens. While aging remains a primary risk factor, recent research has increasingly explored the potential link between cataract development and chronic inflammation (29). Studies have investigated the association between systemic inflammatory markers and the risk of cataract development. Elevated levels of markers such as C-reactive protein (CRP) and interleukin-6 (IL-6) have been implicated in the pathogenesis of cataracts (30). These inflammatory markers may contribute to lens opacification and the progression of cataracts. Chronic inflammation is often accompanied by oxidative stress, and both processes have been related to cataract formation (31). Reactive oxygen species generated during inflammation may contribute to lens damage, accelerating the development of cataracts (30). Antioxidant mechanisms in the lens may become overwhelmed in the presence of chronic inflammation (32). Certain autoimmune conditions associated with chronic inflammation, such as systemic lupus erythematosus, have been linked to an increased risk of cataracts (33). The inflammatory processes inherent in these diseases may contribute to cataractogenesis independently or synergistically with other factors. Lifestyle factors, including diet and smoking, are known to modulate systemic inflammation (34). Emerging research is exploring the role of dietary patterns rich in anti-inflammatory components, such as antioxidants and omega-3 fatty acids, in mitigating the risk of cataracts (35). Conversely, smoking, a pro-inflammatory factor, may exacerbate cataract formation (36).

Several challenges exist in elucidating the intricate relationship between cataracts and chronic inflammation. Variability in study designs, differences in defining and measuring inflammation, and the complex interplay of genetic and environmental factors contribute to the heterogeneity of findings (37). Future research endeavors should prioritize longitudinal studies to establish causality and determine the temporal relationship between chronic inflammation and cataract development. Mechanistic studies exploring the specific pathways through which inflammation influences lens opacification will enhance our understanding of the underlying biology (38). Additionally, targeted interventions, such as anti-inflammatory therapies or lifestyle modifications, may hold promise for preventing or delaying cataract progression in individuals with chronic inflammatory conditions (39). The evolving body of evidence suggests a significant association between chronic inflammation and cataracts. Further research is essential to unravel the complexities of this relationship and pave the way for innovative preventive strategies and therapeutic interventions in the realm of cataract management (40).

Owing to the cross-sectional design, NHANES gathered diet data and cataract information simultaneously. Participants afflicted with cataracts exhibited older age and more comorbidities compared to those without, potentially choosing anti-inflammatory dietary patterns, confounding the causal inference, an aspect unfeasible in cross-sectional studies, leading to a positive association. Cataract surgery served as a proxy for cataract in our study due to the absence of lens examination in NHANES. Analogously, an epidemiological study utilized this method (41). Nevertheless, disparities exist between the approaches. Cataract surgery depends on multiple factors, including grading, visual acuity, clinical decisions, and patients’ choices, potentially influenced by financial conditions (42). Addressing this, we considered financial status as a covariate to mitigate potential confounding effects. Additionally, surgery reflects an advanced cataract stage, limiting examination of earlier-stage lens opacification’s link with DII using NHANES data. Lastly, based upon cataract surgery status, we could not distinguish the cataract types among participants.

In our study, we observed a positive association between the DII and cataract risk, suggesting that diets with higher inflammatory potential may elevate cataract risk. This finding presents an apparent contradiction with studies highlighting the protective effects of anti-inflammatory diets on ocular health. To address this contradiction, it’s essential to consider the complex interplay between dietary components, inflammation, and cataract formation. The discrepancy may stem from variations in dietary intake, nutrient bioavailability, and individual genetic factors that influence the body’s response to dietary inflammation. Moreover, the role of oxidative stress, bridging diet-induced inflammation and cataract development, warrants further investigation. It’s possible that the antioxidant components of certain diets could mitigate the negative effects of a high DII, suggesting the need for a balanced evaluation of diet quality beyond its inflammatory potential. Further research, including longitudinal and intervention studies, is needed to clarify these relationships and guide dietary recommendations for cataract prevention. This nuanced approach acknowledges the complexity of diet-cataract interactions and the importance of comprehensive dietary analysis in public health strategies.

Our study underscores the critical role of dietary patterns in the prevention of cataracts, highlighting the beneficial impact of a diet low in inflammatory potential. Emphasizing the consumption of anti-inflammatory foods, such as fruits, vegetables, whole grains, nuts, and omega-3 fatty acids, can significantly reduce cataract risk. These foods provide essential nutrients and antioxidants like vitamins C and E, lutein, and zeaxanthin, known for their protective effects on eye health. Conversely, reducing intake of pro-inflammatory foods high in saturated fats, trans fats, and refined sugars is advised. From a public health perspective, our findings advocate for the integration of dietary recommendations into health guidelines and public campaigns to educate individuals on the importance of a nutrient-rich diet for cataract prevention. This approach not only supports ocular health but also promotes overall well-being, reinforcing the value of a balanced diet in disease prevention strategies.

This study’s strengths lie in its innovative topic, sizable sample, and comprehensive statistical methods. However, limitations persist. Firstly, being a cross-sectional study precludes causal inferences. Secondly, employing cataract surgery as a proxy introduces latent issues, and thirdly, unaddressed residual confounders.

## 5 Conclusion

In this investigation involving 6,395 participants from a comprehensive nationwide survey, we observed a notably adverse connection between DII and cataract occurrence. This study underscores the risk nature of a Dietary Inflammation Index-aligned diet against cataracts. Additional analyses, including subgroup examination, and non-linear assessments, consistently supported these findings. Nevertheless, substantial prospective studies are necessary to affirm a causal relationship and further validate these conclusions.

## Data availability statement

The original contributions presented in the study are included in the article/supplementary material, further inquiries can be directed to the corresponding authors.

## Ethics statement

The studies involving humans were approved by the Ethics Committee of the Second Affiliated Hospital, Zhejiang University School of Medicine. The studies were conducted in accordance with the local legislation and institutional requirements. The participants provided their written informed consent to participate in this study.

## Author contributions

YZ: Writing – original draft, Methodology, Investigation, Formal analysis. JZ: Writing – review & editing, Visualization, Software, Methodology, Investigation, Data curation. WS: Writing – review & editing. AG: Writing – review & editing. KJ: Writing – review & editing, Validation, Supervision, Project administration, Investigation, Funding acquisition, Data curation, Conceptualization. JY: Writing – review & editing, Supervision, Project administration, Investigation, Funding acquisition, Conceptualization.
